# Multifunctional elastomeric composites based on 3D graphene porous materials

**DOI:** 10.1002/EXP.20230057

**Published:** 2023-10-17

**Authors:** Meichun Ding, Demin Zhao, Rui Wei, Zhenying Duan, Yuxi Zhao, Zeyang Li, Tianhao Lin, Chenwei Li

**Affiliations:** ^1^ School of Chemistry and Pharmaceutical Engineering Shandong First Medical University & Shandong Academy of Medical Sciences Jinan Shandong China; ^2^ Medical Science and Technology Innovation Center Shandong First Medical University & Shandong Academy of Medical Sciences Jinan Shandong China; ^3^ Aix Marseille Univ, CNRS Institut de Chimie Radicalaire (ICR) Marseille France; ^4^ School of The Queen's University of Belfast Joint College China Medical University Shenyang China

**Keywords:** elasticity, graphene aerogels, multifunctionality

## Abstract

3D graphene porous materials (3GPM), which have low density, large porosity, excellent compressibility, high conductivity, hold huge promise for a wide range of applications. Nevertheless, most 3GPM have brittle and weak network structures, which limits their widespread use. Therefore, the preparation of a robust and elastic graphene porous network is critical for the functionalization of 3GPM. Herein, the recent research of 3GPM with excellent mechanical properties are summarized and the focus is on the effect factors that affect the mechanical properties of 3GPM. Moreover, the applications of elastic 3GPM in various fields, such as adsorption, energy storage, solar steam generation, sensors, flexible electronics, and electromagnetic wave shielding are comprehensively reviewed. At last, the new challenges and perspective for fabrication and functionalization of robust and elastic 3GPM are outlined. It is expected that the perspective will inspire more new ideas in preparation and functionalization of 3GPM.

## Graphical abstract

This perspective provides a comprehensive overview of the recent research on 3D graphene porous materials (3GPM) with excellent mechanical properties. It analyzes the factors that affect the mechanical behaviour of 3GPM and examines their diverse applications in various fields, such as adsorption, energy storage, solar steam generation, sensors, flexible electronics, and electromagnetic wave shielding. Finally, it outlines the new challenges and opportunities for the fabrication and functionalization of robust and elastic 3GPM.

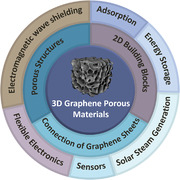

## 1 INTRODUCTION

Three‐dimensional porous materials (3PM) (aerogels, sponges, and foams), which have large porosity, low density, excellent mechanical properties, high conductivity, and excellent mechanical properties, have been demonstrated in a wide range of applications.^[^ ^1^, ^2^, ^3^ ^]^ Because 2D materials have a high aspect and strong interactions between 2D materials, 2D materials are more suitable for synthesis of 3PMs than 1D and 0D materials.^[^ ^4^ ^]^ Since 2D graphene material was first prepared in 2004, its unique electrical, mechanical, and thermal properties have drawn wide attention.^[^ ^5^, ^6^, ^7^ ^]^ In order to apply the properties at the nanoscale to macroscopic applications, assembly of 2D graphene sheets into 3D networks are required. Graphene oxide (GO) is a functionalized graphene material which can be synthesized by using a Hummers’ method.^[^ ^8^, ^9^ ^]^ GO is most widely used in the construction of graphene network, its oxygen‐containing groups impart good processability for assembly into a 3D graphene porous material (3GPM).^[^ ^10^ ^]^ Previously reported 3GPM generally have fragile network structure, which limiting the widespread use of the 3GPM.^[^ ^11^ ^]^ Therefore, the construction of a robust graphene network structure is critical for the multifunctional 3GPM.

Due to a balance between electrostatic repulsion and Van der Waals interaction, the GO can uniformly disperse in the solution.^[^ ^12^ ^]^ The balance will be broken to form hydrogel by some methods (such as adding crosslinker or reducing agent, changing pH of mixed solution, ultrasonicating the mixed solution, and hydrothermal reduction).^[^ ^13^ ^]^ During gelation process, 2D graphene sheets are linked to form 3D graphene network, followed by some drying methods (such as freeze‐drying, supercritical‐drying, air‐drying), 3GPM are obtained.^[^ ^10^ ^]^ For instance, simple chemical reduction of GO solution is often used to fabricate 3GPM. Na_2_S, Vitamin C, NaHSO_2_, hydroiodic acid, and sodium ascorbate could be used as the reducing agents.^[^ ^14^ ^]^ The densities of 3GPM can be changed from 0.16 to 96 mg cm^−3^ by manipulating drying methods and the concentration of GO solutions.^[^ ^14^ ^]^ However, these reported 3GPM generally exhibit brittle mechanical properties, and the graphene network is easily broken under working conditions.^[^ ^11^ ^]^ It is challenging to fabricate the 3GPM with low density and high mechanical property. The mechanical properties of 3GPM are related to factors such as 2D building blocks, connection of 2D materials, and graphene porous structures. To balance these factors is very important for building the 3GPM with robust network. Herein, we summarize the latest representative research in the fabrication and applications of robust 3GPM and the influence factors of robust 3GPM, in order to promote the large‐scale fabrication and application of 3GPM and inspire new ideas for the functionalization of 3GPM.

Herein, we summarize the recent research of 3GPM with excellent mechanical properties and focus on the effect factors that affect the mechanical properties of 3GPM.

At last, we outline the new challenges and perspective for fabrication and functionalization of robust and elastic 3GPM. It is expected that our short review will inspire more new ideas in preparation and functionalization of 3GPM.

## 2 SYNTHESIS OF 3GPM

### 2.1 2D Building blocks

**FIGURE 1:**
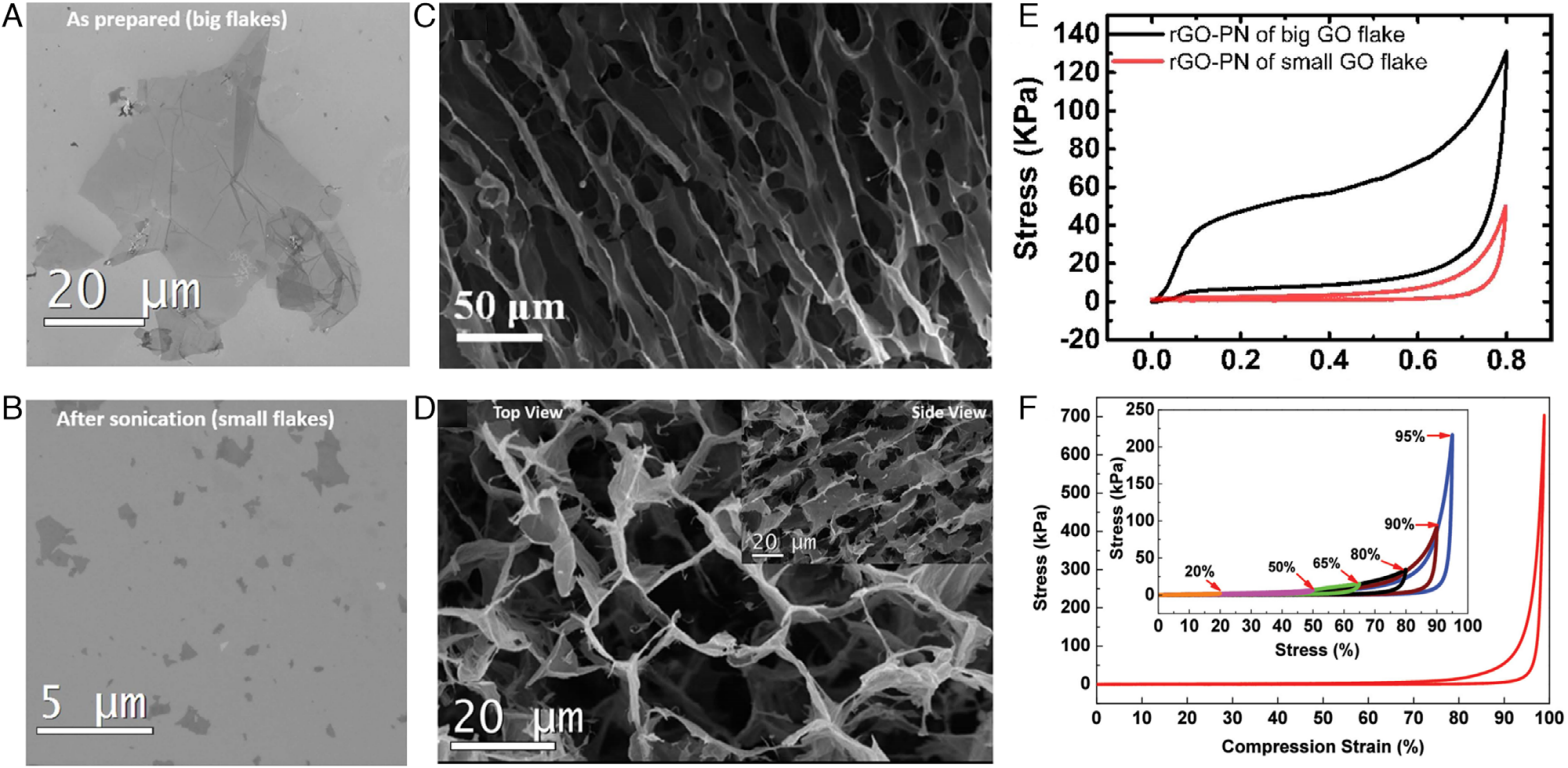
SEM image of A, large and B, small GO sheets. Reproduced with permission.^[^ ^15^ ^]^ Copyright 2015, Springer Nature. SEM image of 3GPM prepared by C, large and D, small GO sheets. Reproduced with permission.^[^ ^15^ ^]^ Copyright 2015, Springer Nature. E, Compressive stress–strain curves of 3GPM (∼11 mg cm^−3^) produced by large and small GO sheets. Reproduced with permission.^[^ ^15^ ^]^ Copyright 2015, Springer Nature. F, The compression test with the strain up to 99%. Reproduced with permission.^[^ ^16^ ^]^ Copyright 2016, Wiley‐VCH GmbH. Inset: the stress–strain curve of 3GPM at different compressive strains.

GO sheets are the basic unit of graphene network and their characteristics determine the mechanical properties of 3GPM. Ni et al. used GO sheets with different size (∼2 and 20 μm) (Figure 1A,B) to fabricate 3GPM and studied the mechanical properties of 3GPM.^[^ ^15^ ^]^ The SEM images of network structures of 3GPM prepared by GO sheets with different size are shown in Figure 1C,D, respectively. The 3GPM fabricated by GO sheets (>20 μm) exhibited the porous graphene structures (Figure 1C), which are similar to other graphene based porous networks. The 3GPM produced with small GO sheets exhibited a much more fragmented structure (Figure 1D). The compressive tests were used to analyze the effect of GO sheet size on the mechanical performance of 3GPM prepared by GO sheets with different size. As shown in Figure 1E, the 3GPM produced by small GO sheets exhibited smaller compressive stress, Young's modulus, and poor elasticity. In contrast, the 3GPM prepared by large GO sheets were much stronger and showed excellent elastic resilience after deformation. Zhang et al. also used large GO sheets (∼2000 μm^2^) to fabricate the 3GPM with excellent reversible compressibility of up to 99% (Figure 1F).^[^ ^16^ ^]^ Therefore, the GO sheets size plays a crucial role in the mechanical performance of 3GPM.

### 2.2 Connection of graphene sheets

Due to the 2D graphene sheets are connected to form 3D network, it is effective strategy to fabricate elastic and robust 3GPM by enhancement the connection of graphene. 3PM was prepared by using GO sheets and carbon nanotubes (CNT) as the two synergistic building blocks.^[^ ^17^ ^]^ Because of the synergistic effect between CNT and graphene, the graphene/CNT porous materials possessed the ultralow density and excellent elasticity at −196 and 300°C. We demonstrated a new method to fabricate elastic 3GPM by using polyacrylamide to enhance the connection of graphene sheets.^[^ ^18^ ^]^ While only ∼1 wt% of polyacrylamide were present in 3GPM, the compressive stresses of 3GPM were improved by 50∼100% compared with 3GPM without polyacrylamide. After the vacuum‐drying and air‐drying, the robust 3D graphene network could resist the destruction of capillary force and kept the network structures intact. More importantly, vacuum‐drying and air‐drying were more convenient and energy saving than freeze‐drying and supercritical‐drying techniques. Recently, we have successfully fabricated elastic 3GPM^[^ ^19^ ^]^ through a thermal treatment of the 3GPM prepared in our previous work.^[^ ^18^ ^]^ After thermal treatment, the functional groups (such as hydroxyl, carboxyl, carbonyl, and so on) on the graphene were eliminated and the sp^2^ regions of graphene were restored, resulting in the enhanced interaction between graphene sheets. Because of robust cell walls, 3GPM can retain the substantial elastic resilience after a loading of 100.00 kg. The 3GPM showed the superelasticity under maximum strain of ∼99.8% and ultimate stress of above 1000 MPa, which is the best reported result compared with previously reported porous materials.^[^ ^20^, ^21^, ^22^, ^23^, ^24^, ^25^, ^27^ ^]^

### 2.3 Porous structures

Graphene network structure is another important factor which affects the mechanical properties of 3GPM. 2D GO assembled into 3D network with disordered structure, which was attributed to the lack of effective control during the construction of 3D graphene network process. Therefore, these reported 3GPM generally show brittleness and the graphene network is easily broken during deformations. How to control the graphene network structure is one of crucial factors in the fabrication of the robust 3GPM.

**FIGURE 2:**
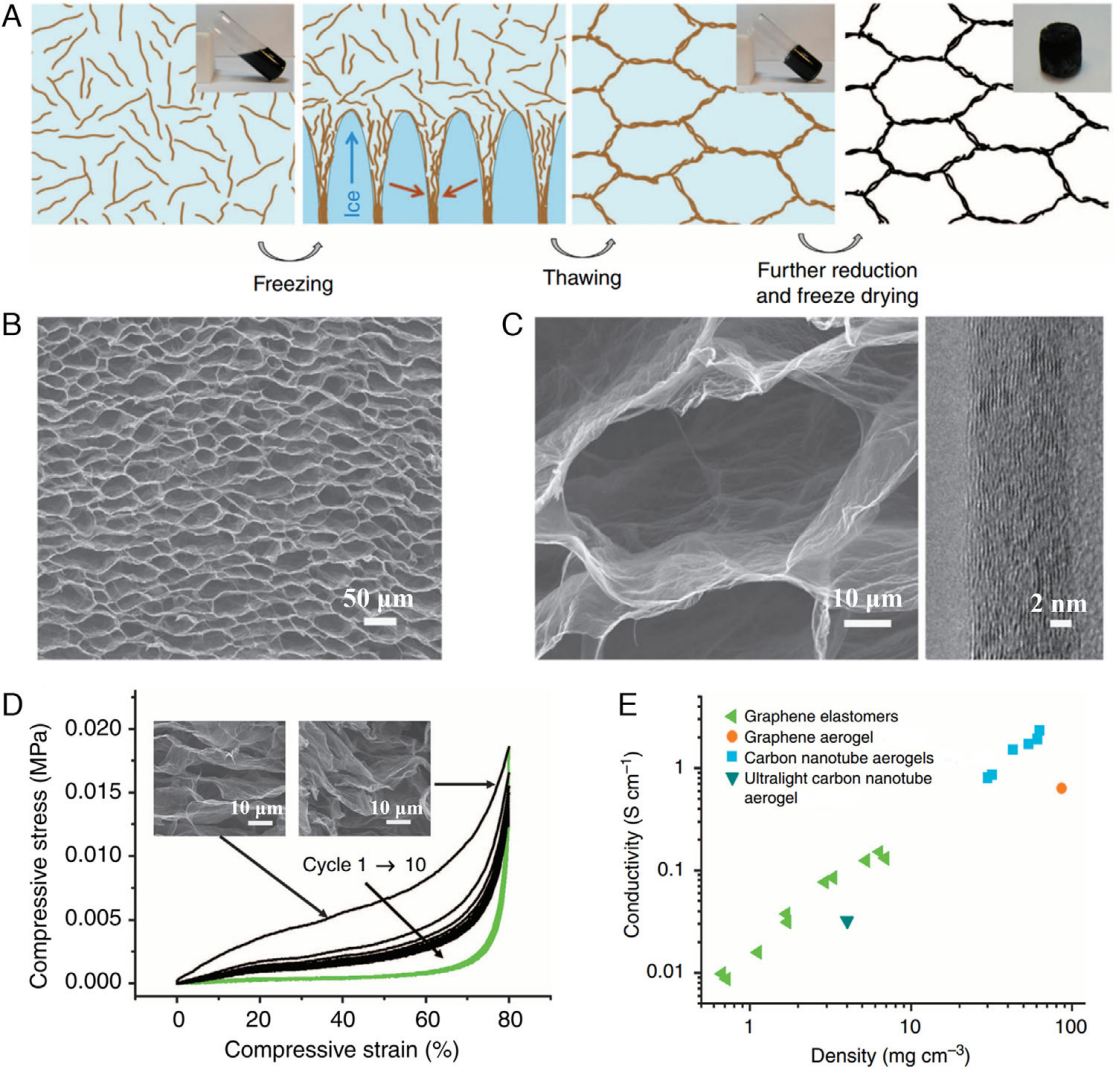
A, Schematic illustrations for the preparation of 3GPM with biomimetic hierarchical structure by using a two‐steps method. Reproduced with permission.^[^ ^28^ ^]^ Copyright 2012, Springer Nature. B, SEM image of 3GPM. Reproduced with permission.^[^ ^28^ ^]^ Copyright 2012, Springer Nature. C, SEM (left) and TEM (right) image of 3GPM with a density of 5.10 mg cm^−3^. Reproduced with permission.^[^ ^28^ ^]^ Copyright 2012, Springer Nature. D, Compressive stress–strain curves of 10 cycles of loading and unloading. Reproduced with permission.^[^ ^28^ ^]^ Copyright 2012, Springer Nature. The inserts show the SEM images of 3GPM under compression at different strains. E, Electrical conductivity versus densities of 3GPM and other carbon‐based porous materials. Reproduced with permission.^[^ ^28^ ^]^ Copyright 2012, Springer Nature.

The 3GPM with hierarchical structure was prepared by using two‐step method (Figure 2A).^[^ ^28^ ^]^ First, the partially reduced GO (p‐GO) solution was directional frozen in a dry ice bath. Upon directional freezing process, the p‐GO sheets aligned along the directionally grown ice crystal and formed ordered 3D network structures due to the interaction between the p‐GO sheets. Second, the p‐GO composite was further reduced and the 3GPM was obtain after freeze‐drying. During the further reduction process, the interactions between graphene sheets were enhanced, resulting in the robust 3D graphene network. 3GPM exhibited porous structures and the cell walls composed of oriented layer structure (Figure 2B,C). Benefiting from the ordered network structures, 3GPM exhibits a low density (0.56∼6.62 mg cm^−3^) and excellent resilience simultaneously. The 3GPM with the density of ∼5.1 mg cm^−3^ have the ultimate stress values higher than 2–9 orders of those of other porous materials under 80% compressive strain. After compression tests at 80% strain for 10 cycles, the 3GPM can retain the porous structure intact (Figure 2D). Moreover, the porous structures and cell walls composed of stacked graphene sheets facilitate electron transmission, leading to the enhanced electrical performance of 3GPM (Figure 2E).

**FIGURE 3:**
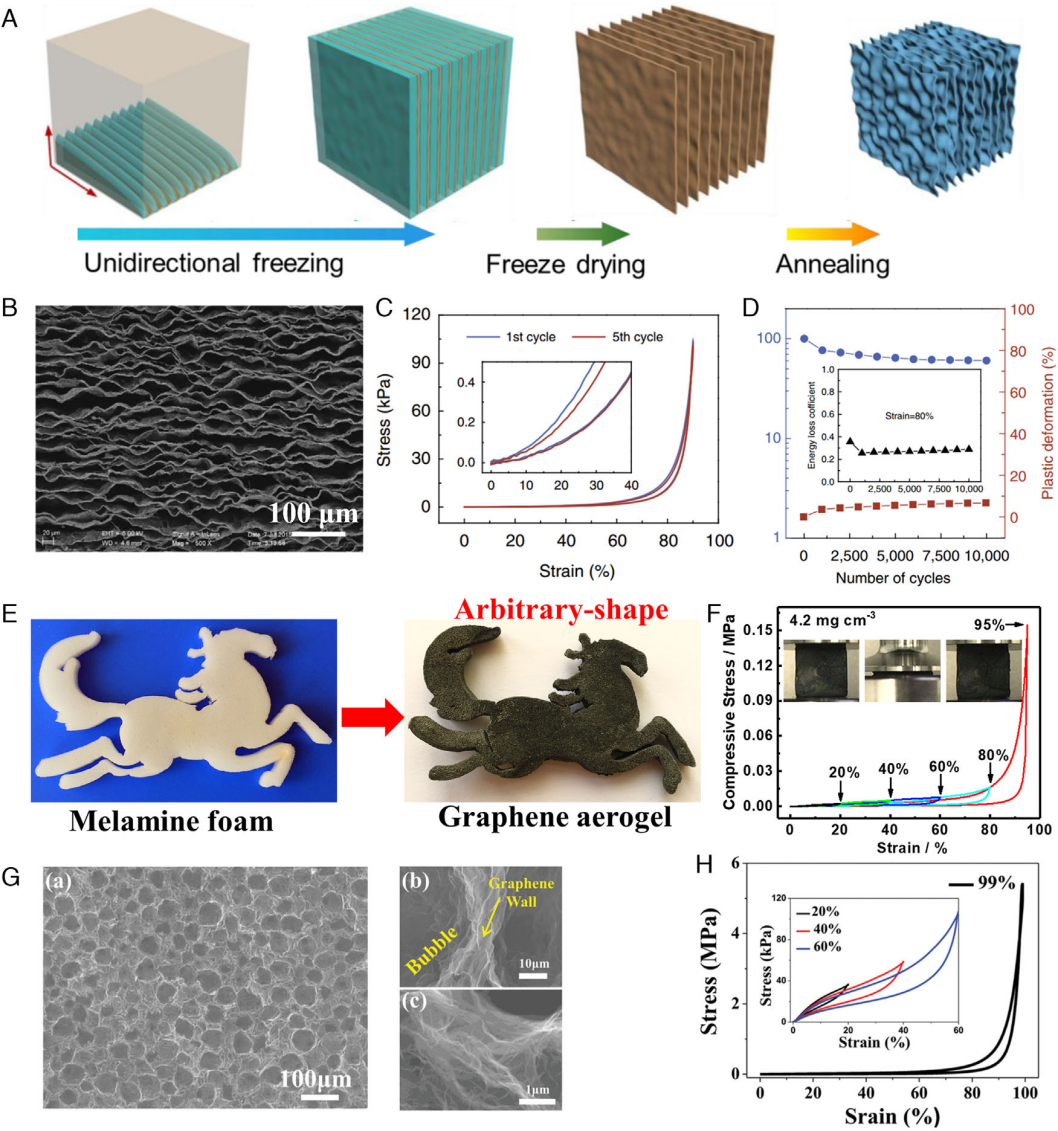
A, Schematic illustrations for the preparation of 3GPM with lamellar multi‐arch microstructure by using bidirectional freezing method. Reproduced with permission.^[^ ^29^ ^]^ Copyright 2016, Springer Nature. B, SEM image (top view) of 3GPM shows the lamellar multi‐arch microstructure. Reproduced with permission.^[^ ^29^ ^]^ Copyright 2016, Springer Nature. C, Stress–strain curves of 3GPM. Reproduced with permission.^[^ ^29^ ^]^ Copyright 2016, Springer Nature. D, Elastic strength, plastic deformation and energy loss coefficient during 10,000 cycles at 80% strain. Reproduced with permission.^[^ ^29^ ^]^ Copyright 2016, Springer Nature. E, Fabrication of 3GPM with arbitrary shape by using melamine foam as sacrificial skeleton. Reproduced with permission.^[^ ^30^ ^]^ Copyright 2018, Wiley‐VCH GmbH. F, The stress–strain curves of 3GPM (4.2 mg cm^−3^) at a maximum compressive strain of 95%. Reproduced with permission.^[^ ^30^ ^]^ Copyright 2018, Wiley‐VCH GmbH. G, SEM images of 3GPM with bubble‐like cell structure at different magnifications. Reproduced with permission.^[^ ^31^ ^]^ Copyright 2016, Wiley‐VCH GmbH. H, Stress–strain curves at different compressive strains. Reproduced with permission.^[^ ^31^ ^]^ Copyright 2016, Wiley‐VCH GmbH.

There are other strategies to fabricate robust and elastic 3GPM by controlling the graphene network structures. Gao et al. fabricated a graphene‐carbon porous material with lamellar multi‐arch microstructure by using bidirectional freezing method (Figure 3A).^[^ ^29^ ^]^ Due to the unique lamellar multi‐arch network structure, the graphene‐carbon porous material shows superelasticity and superior fatigue resistance (Figure 3B,C). The graphene‐carbon porous material showed ∼7% reduction in height after 10,000 compression cycles at 80% strain (Figure 3D). We fabricated the elastic, arbitrary‐shaped, and durable 3GPM by using commercially available melamine foam as sacrificial skeleton (Figure 3E).^[^ ^30^ ^]^ Since the skeleton of melamine foam effectively prevents serious accumulation of GO sheets during the reduction process, 3GPM showed ordered network structures with several tens of micrometer. 3GPM of 4.2 mg cm^−3^ exhibited high elasticity with compressive stress of 0.556 MPa and compressive strain of 95% (Figure 3F). Lv et al. reported the air‐bubble template method to fabrication of robust 3GPM without involving the organic droplets.^[^ ^31^ ^]^ SEM images of 3GPM demonstrate unique air bubble‐like cell structure with the cell dimension of about 60 μm (Figure 3G). The 3GPM showed high ultimate stress of ∼5.4 MPa at maximum strain of ∼99% and retained resilience after 1000 compression cycles (Figure 3H).

## 3 APPLICATIONS

3GPM composed of graphene inherits the unique performance of graphene, resulting in 3GPM could provide new features that conventional porous materials cannot offer. What's more, the high mechanical properties of 3GPM expand their importance in many applications.

### 3.1 Adsorption

**FIGURE 4:**
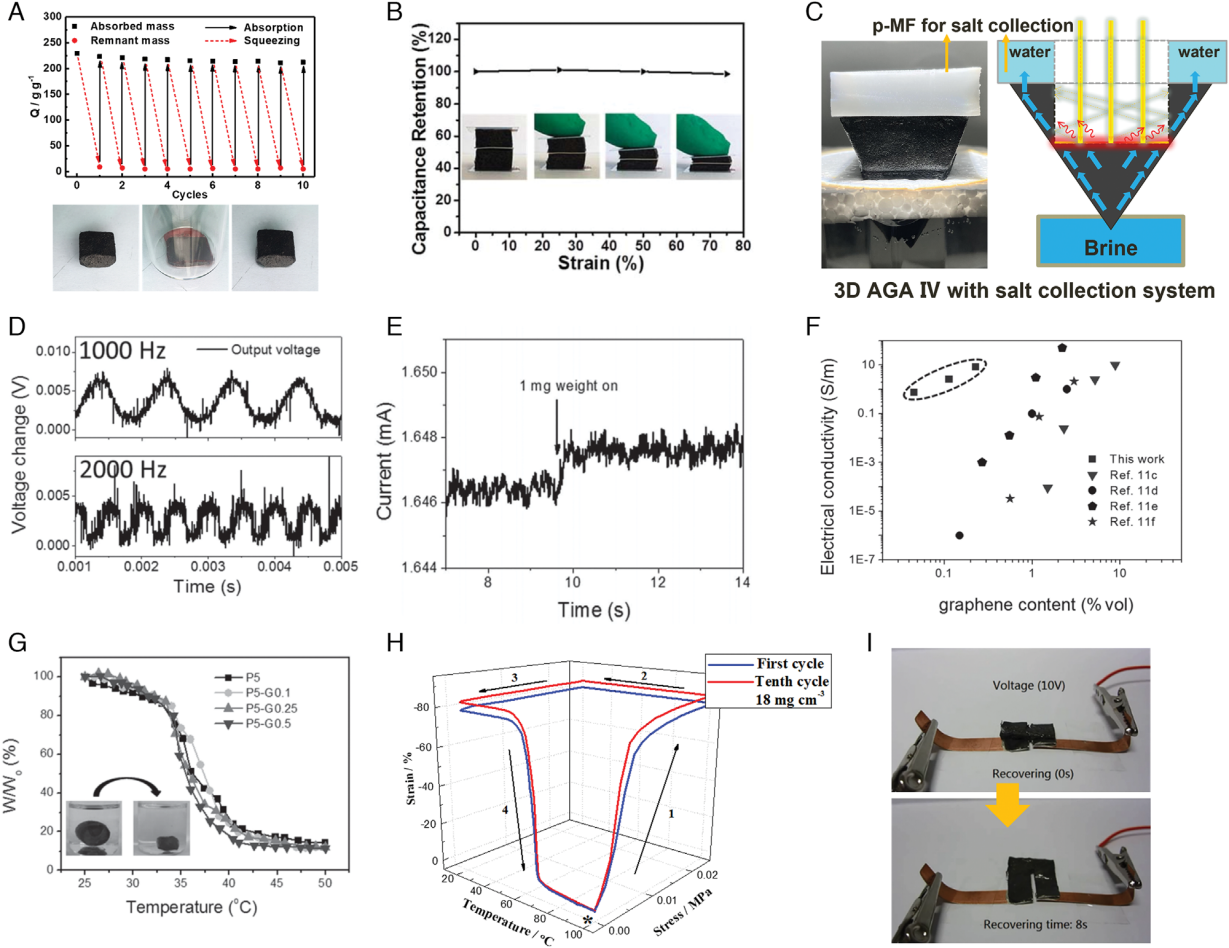
A, Squeezing was used to recycle the 3GPM for adsorption of ethanol. Snapshots of the process for recycling 3GPM via squeezing. Reproduced with permission.^[^ ^30^ ^]^ Copyright 2018, Wiley‐VCH GmbH. B, Capacitance retention of a compressible supercapacitor fabricated by 3GPM at different strains of 0%, 25%, 50% and 75%. Reproduced with permission.^[^ ^37^ ^]^ Copyright 2017, The Royal Society of Chemistry. C, Photograph and schematic illustration of 3D assembled clay/graphene aerogel for continuous solar desalination. Reproduced with permission.^[^ ^48^ ^]^ Copyright 2018, Wiley‐VCH GmbH. D, High frequency piezoresistive responses of 3GPM. Reproduced with permission.^[^ ^49^ ^]^ Copyright 2016, Wiley‐VCH GmbH. The electrical output over time with two input frequencies (1000 and 2000 Hz). E, The 3GPM of 0.54 mg cm^−3^ detecting ultralow pressure (1 mg weight on an area of 113 mm^2^ is equivalent to 0.082 Pa). Reproduced with permission.^[^ ^49^ ^]^ Copyright 2016, Wiley‐VCH GmbH. F, Electrical conductivity of graphene/PNIPAM hydrogels and other reduced graphene oxide‐based materials. Reproduced with permission.^[^ ^51^ ^]^ Copyright 2014, Wiley‐VCH GmbH. G, Deswelling ratio of PNIPAM and graphene/PNIPAM hydrogels as function of temperature. Reproduced with permission.^[^ ^51^ ^]^ Copyright 2014, Wiley‐VCH GmbH. Inserts are the photo images of graphene/PNIPAM hydrogel before and after deswelling. H, Thermotropically induced shape‐memory behavior of TPI/graphene foam (18 mg cm^−3^) through 10 cycles of compression and recovery. Reproduced with permission.^[^ ^18^ ^]^ Copyright 2016, Wiley‐VCH GmbH. I, The deployable TPI/graphene foam rapidly recovered to its original shape in ≈8 s, when the DC voltage (10 V) was applied. Reproduced with permission.^[^ ^18^ ^]^ Copyright 2016, Wiley‐VCH GmbH.

Due to the hydrophobicity and high porosity, the 3GPM showed excellent adsorption capability for organic solvents and oils.^[^ ^32^ ^]^ In practical applications, the reusability of 3GPM and collection of organic solvents are critical because most organic solvents are valuable or noxious.^[^ ^33^ ^]^ For 3GPM with brittle mechanical properties, the absorbent could be collected by using heating and condensation.^[^ ^34^ ^]^ But this method is not only complicated, but also difficult to collect high boiling point solvents. For robust and elastic 3GPM, squeezing is an easy way to recovering valuable organic solvents. The elastic graphene network endows 3GPM with excellent adsorption performance stability (Figure 4A).^[^ ^30^ ^]^ Based on the elastic graphene network, the oil‐water separation performance (42,402 L m^−2^ h^−1^) was enhanced by changing the porosity and surface roughness of 3GPM by introducing SiO_2_ nanoparticles and PVDF nanofibers.^[^ ^35^ ^]^ Thanks to excellent thermal stability of 3GPM, the combustion is an ideal method to remove adsorbed toxic or low‐value solvents. Zhou et al. prepared a lamellar interpenetrated graphene/carbon network, which showed high adsorption capacity and recyclability (the absorption capacity remains ∼90% after cyclic absorption/combustion tests).^[^ ^36^ ^]^

### 3.2 Energy storage

Recently, with the development of wearable and portable electronics, lightweight and compressible supercapacitors become a new research focus. We demonstrated a strategy for fabricating a 3D nitrogen‐doped/graphene porous material with excellent elasticity and stable electrochemical property.^[^ ^37^ ^]^ An all‐solid supercapacitor is fabricated with 3D nitrogen‐doped/graphene porous material as the electrode, which showed a specific capacitance (150 F g^−1^, 0.3 A g^−1^) and stable electrochemical property (at 75% strain) (Figure 4B). Li et al. prepared covalent organic frameworks (COFs)/reduced graphene oxide (rGO) aerogels as active material of supercapacitor by using a hydrothermal method. The COF/rGO aerogels shows a high specific capacitance (269 F g^−1^, 0.5 A g^−1^) and stable electrochemical property (over 5000 cycles).^[^ ^38^ ^]^ Therefore, the 3GPM have great potential in flexible/elastic wearable devices.

Elastic 3GPMs also exhibit great potential as an electrode material for rechargeable batteries, including lithium‐ion, sodium‐ion, and zinc‐ion batteries. Its remarkable properties, such as high porosity, large surface area, excellent mechanical stability, and good electrical conductivity, enable fast ion transport, high specific capacity, and long cycle life in batteries. The remarkable properties, such as high porosity, large surface area, excellent mechanical stability, and good electrical conductivity, enable fast ion transport, high specific capacity, and long cycle life in batteries.^[^ ^39^, ^40^, ^41^ ^]^ Moreover, the use of elastic 3GPMs help mitigate electrode degradation by accommodating the volume changes that occur during charge and discharge processes, thereby further enhancing battery performance.^[^ ^40^ ^]^ For instance, when employed as an anode material in a lithium‐ion battery, 3GPM demonstrated an impressive reversible capacity of 800 mAh g^−1^ after 100 cycles at a current density of 100 mA g^−1^.^[^ ^39^ ^]^ Similarly, as a cathode material in a sodium‐ion battery, it exhibited a high initial capacity of 160 mAh g^−1^ and maintained a stable capacity retention of 90% after 200 cycles at a current density of 50 mA g^−1^.^[^ ^40^ ^]^ Furthermore, in the case of a zinc‐ion battery, 3GPM used as the cathode material displayed a significant specific capacity of 300 mAh g^−1^ and demonstrated excellent cycling stability with over 500 cycles at a current density of 100 mA g^−1^.^[^ ^41^ ^]^

### 3.3 Solar steam generation

Solar steam generation is the most promising method to obtain fresh water.^[^ ^42^, ^43^, ^44^, ^45^ ^]^ Zhang et al. used the antifreeze‐assisted freezing technique to fabricate the 3GPM with vertically aligned structure.^[^ ^46^ ^]^ The 3GPM with high porosity, excellent photothermal conversion performance, and broadband absorption (from 250 to 2500 nm) are the ideal materials for solar steam generation. The 3GPM with vertically aligned structure showed the high evaporation rate of 1.62 kg m^−2^ h^−1^ and superb steam generation efficiency of 86.5% under one sun irradiation. In working conditions, the robust graphene network maintained structural intact and evaporation performance under dynamic fluid flow and volumetric stress. Moreover, the robust graphene network plays an important role in the evaporation performance. Li et al. prepared vertically aligned RGO aerogel with numerous vertical channels for solar steam generation.^[^ ^47^ ^]^ During the compression of vertically aligned RGO aerogel process, the evaporation enthalpy of water decreased significantly due to the increase of the intermediate water ratio, resulting in an evaporation performance of 3.39 kg m^−2^ h^−1^ under 1 solar irradiation. Recently, Ding et al. prepared a 3D assembled clay/graphene aerogels for continuous solar desalination. The 3D assembled clay/graphene aerogels achieved a high evaporation rate of 4.11 kg m^−2^ h^−1^ in 20 wt% brine under continuous illumination (one sun) for 36 h without salt precipitation (Figure 4C).^[^ ^48^ ^]^ Therefore, the 3GPM have great potential for high‐efficient and stable solar‐driven vaporization and desalination.

### 3.4 Sensors

During the compression of 3GPM, the change in contact area of graphene sheets leads to changes in their resistance. The elastic 3GPM can be used as quasi‐static pressure and strain sensors through detecting changes in the electrical resistance of 3GPM during mechanical compression. Since the compression process of 3GPM has little effect on its piezoresistive behavior, 3GPM has great potential to provide an electronic response to the compression process. Because of excellent conductivity and elastic network structure, the compression process of 3GPM has little effect on its piezoresistive behaviour. Recently, Qiu et al. used the robust 3GPM as a sensor system to detect the dynamic forces ranging from quasi‐static to 2000 Hz (Figure 4D).^[^ ^49^ ^]^ Moreover, 3GPM with the ultralow density (0.54 mg cm^−3^) showed the ultrahigh sensitivity (10 kPa^−1^) and even a minimum pressure of 0.082 Pa could be detected (Figure 4E). Thanks to the robust graphene network and stable current changes under large deformations or bends, the 3GPM could be used as a wearable pressure/strain sensor for detecting human motions.^[^ ^30^ ^]^ Based on RGO/poly(3,4‐ethylenedioxythiophene) (PEDOT): poly‐(styrenesulfonate) (PSS) aerogel, Kong et al. proposed a hair‐epidermis‐dermis hierarchical structure to reconcile this contradiction between high sensitivity and a wide linear range.^[^ ^50^ ^]^ The RGO/PEDOT:PSS aerogel enables an electronic skin sensor with a linear sensing range of up to 30 kPa without sacrificing high sensitivity (137.7 kPa^−1^). Moreover, the electronic skin sensor also exhibits a low detection limit (1.1 Pa), fast responsiveness (∼80 ms), and excellent stability and reproducibility (over 10 000 cycles).

### 3.5 Flexible electronics

The robust and elastic 3GPM also make it possible to prepare multifunctional graphene composites. Qiu et al. used a robust and ultralight 3GPM as the skeleton to fabricate the graphene/poly(*N*‐isopropylacrylamide) (PNIPAM) hydrogels with stimuli‐responsiveness and high electrical conductivity.^[^ ^51^ ^]^ Due to the elastic graphene skeleton, the graphene/PNIPAM hydrogel could recover its original shape after cyclic compression tests (up to 90% strain). The graphene/PNIPAM hydrogel showed a conductivity of ∼10 S m^−1^ when graphene content of ∼0.23 vol.%, which was higher than 1–7 orders of magnitude compared with those of other graphene‐based composites and hydrogels with similar graphene loadings (Figure 4F). Compared to PNIPAM hydrogel, the graphene/PNIPAM hydrogels exhibited similar deswelling ratios (∼90%) and lower critical solution temperature (35°C) (Figure 4G). The graphene skeleton had little effect on the esponsive function of the graphene/PNIPAM hydrogels, which was in contrast to other PNIPAM hybrid hydrogels whose responsive behaviour was restricted by the randomly dispersed nanofillers. The trans‐1,4‐polyisoprene (TPI)/graphene hybrid foams were fabricated by using the elastic 3GPM as the skeleton.^[^ ^18^ ^]^ The resulting ultralight (18–56 mg cm^−3^) TPI/graphene aerogels exhibited superior thermotropic shape memory performances. The recovering ratios of TPI / graphene aerogels (18 mg cm^−3^) were above 99% after the 10 cycles of loading and unloading, while the value of recovering ratio for other shape‐memory polymer foam was only 63% after the first cycle (Figure 4H). Due to the excellent conductivity of 3D graphene skeleton, TPI/graphene aerogels also showed electrically actuating shape‐memory effect. The deployable samples rapidly recovered their own shape in a voltage of 10 V and a recovery time of 8 s (Figure 4I). Due to the stretchable and robust graphene network, Guo et al. prepared the graphene/shape memory polymer aerogels with a fast response of ∼175 mm s^−1^, large deformation of ∼100%, and a wide response bandwidth of 0.1–20 Hz.^[^ ^52^ ^]^ Therefore, the ultrafast response of graphene/hape memory polymer aerogels confers extensive uses in micro‐oscillators, artificial muscles, actuators, soft robotics, and so on.

### 3.6 Electromagnetic wave shielding

Graphene‐based materials are the most promising candidates for addressing electromagnetic wave pollution due to their superior structure and enhanced electromagnetic properties. Graphene‐based porous materials with 3D porous networks have been drawn wide attention for electromagnetic wave shielding materials due to low‐density, high electric conductivity, and excellent mechanical properties. The electromagnetic wave absorption property of 3GPM can be improved by turning the porous structure to obtain better impedance matching. The electromagnetic wave absorption performances of 3GPM depend on the conductive network.^[^ ^53^ ^]^ Ma et al. prepared a lightweight 3GPM with controlled density for high‐efficient electromagnetic performance.^[^ ^54^ ^]^ The conductive pathway and dielectric properties can be easily tuned by manipulating the concentration of GO solutions. The resulting 3GPM with a low‐density (4.5 mg cm^−3^) showed a reflection loss (RLmin) of −50 dB at a thickness of 1.14 mm. Moreover, 3GPM can be developed as a responsive material with compression‐dependent electromagnetic absorption performance due to the elastic network. Zhang et al. tuned the electromagnetic wave absorption properties of elastic 3GPM by changing the compressive strain.^[^ ^55^ ^]^ The 3GPM showed an RLmin of −35 dB at thickness of 1 mm and an effective absorption bandwidth of 64.5 GHz under compressive strain of 90%.

## 4 PERSPECTIVE

In summary, the increasing attention demonstrate the enormous potential and practical possibility of elastic and robust 3D graphene porous materials for varied applications. Much progress has been achieved on fabrication of 3GPM with excellent elastic resilience, such as controlling the graphene network structures and rationally enhancing the structural strength. Fabrication of elastic and robust 3GPM extends the application of graphene porous materials including pressure/strain sensor, adsorption, compressible solid‐state supercapacitor, solar steam generation device and so on. There are still some difficulties and challenges to overcome. First, how to precisely control the network structure of 3GPM, such as the arrangement of cell, cell size, and graphene layers of the cell walls. Most of reported 3GPM generally have a randomly structured network of broad cell size distribution which results in the brittle mechanical properties. Using 3D printing technology to fabricate 3GPM with pre‐designed network structures is a possible solution. At present, the resolution of the graphene network structures produced by 3D printing technique cannot meet the requirement,^[^ ^56^, ^57^ ^]^ demanding the development of the 3D printing technique with higher resolution. Second, how to further enhance the graphene network strength of 3GPM. Enhancement of the cross‐linking between the graphene sheets to form robust graphene network by adding a crosslinking agent. The findings gained in 3GPM may open a new avenue to fabricate new porous materials derived from other 2D materials such as MXene, MoS_2_, C_3_N_4_, and BN.

## CONFLICT OF INTEREST STATEMENT

The authors declare that they have no known competing financial interests or personal relationships that could have appeared to influence the work reported in this paper.
